# Establishing best practices in large language model research: an application to repeat prompting

**DOI:** 10.1093/jamia/ocae294

**Published:** 2024-12-04

**Authors:** Robert J Gallo, Michael Baiocchi, Thomas R Savage, Jonathan H Chen

**Affiliations:** Center for Innovation to Implementation, VA Palo Alto Health Care System, Menlo Park, CA 94025, United States; Department of Health Policy, Stanford University, Stanford, CA 94305, United States; Department of Epidemiology and Population Health, Stanford University, Stanford, CA 94305, United States; Division of Hospital Medicine, Stanford University, Stanford, CA 94305, United States; Division of Hospital Medicine, Stanford University, Stanford, CA 94305, United States; Stanford Center for Biomedical Informatics Research, Stanford University, Stanford, CA 94304, United States; Clinical Excellence Research Center, Stanford University, Stanford, CA 94305, United States

**Keywords:** large language model, peer review, multilevel analysis

## Abstract

**Objectives:**

We aimed to demonstrate the importance of establishing best practices in large language model research, using repeat prompting as an illustrative example.

**Materials and Methods:**

Using data from a prior study investigating potential model bias in peer review of medical abstracts, we compared methods that ignore correlation in model outputs from repeated prompting with a random effects method that accounts for this correlation.

**Results:**

High correlation within groups was found when repeatedly prompting the model, with intraclass correlation coefficient of 0.69. Ignoring the inherent correlation in the data led to over 100-fold inflation of effective sample size. After appropriately accounting for this issue, the authors’ results reverse from a small but highly significant finding to no evidence of model bias.

**Discussion:**

The establishment of best practices for LLM research is urgently needed, as demonstrated in this case where accounting for repeat prompting in analyses was critical for accurate study conclusions.

## Background

While large language models (LLMs) have shown promise for many medical applications, the rapid evolution of the field may have outpaced the development of robust research practices. For example, it is often necessary to evaluate stability and uncertainty in model responses, as LLMs have randomness built into their outputs. Repeat prompting can therefore be helpful to capture variation in model outputs,^1^^,^^2^ with studies in a number of medical journals utilizing this method.^3–7^ Adding additional observations for the same or similar prompts can be trivial from the researcher’s perspective given ease of repeatedly prompting the model, tempting researchers to increase sample size by orders of magnitude. However, this practice leads to additional methodological considerations that may not be familiar to researchers, with implications for study conclusions if ignored.

Model outputs to repeat prompting are likely to be strongly correlated leading to violations of the independence assumption for many statistical tests.^8–10^ This is analogous to prompting a single individual to answer a survey question 250 times in a manner that they do not remember their previous responses, but then analyzing the data using methods that assume the responses came from 250 different individuals. We would expect responses from a single individual to be highly correlated with less variation than responses from 250 individuals, leading to artificially small CIs and *P*-values.^11^ Despite this intuition, others have suggested that LLM responses to repeat prompting might rather represent independent samples given model complexity and randomness, with further evaluation needed to establish consensus.^12^

## Objective

In this study, we sought to determine the importance of accounting for correlation in repeat prompting of LLMs and its effect on research conclusions in order to explore best practices. We use data from a study on affiliation bias in peer review of medical abstracts by an LLM as an instructive example.^3^

## Methods

### Study design

The data utilized for the study have been described previously, with GPT-3.5 prompted to review and decide on acceptance for 30 abstracts.^3^ Each abstract was duplicated and attached with 30 different university affiliations categorized into 3 tiers, for a total of 900 abstract-affiliation combinations.^3^ The university affiliations were obtained by prompting ChatGPT to provide examples of 10 “top-tier”, 10 “mid-tier”, and 10 “low-tier” medical research universities so that the tiers used in the study would best reflect any potential bias encoded in the model. The authors repeatedly prompted the model 250 times for each abstract-affiliation combination, for a total sample of 225 000 observations. The original authors used a difference in proportions test to evaluate the hypothesis that the model would be biased to more likely accept abstracts attached with higher tier affiliations.

We also provide an example in the Supplementary Material where repeat prompting was performed with variations in the prompt, which may lead to more randomness, and therefore less correlation, in outputs. For this example, we use data from our prior study evaluating 5 different prompting strategies for diagnostic reasoning.^13^ That previously published study compared diagnostic accuracy across prompting strategies, but for this purpose we compare the correlation of outputs from the variations in prompting.

### Statistical analysis

In this secondary analysis, we use a mixed-effects logistic regression model to account for correlation in repeat prompts compared to a simple logistic regression model that assumes independence of observations, similar to the analysis used in the original study. Random effects for abstract and affiliation were included in the mixed-effects model to account for repeat prompting, as considering either alone would not capture correlation at the abstract-affiliation combination level. Although statistical models should be constructed to fit the data-generating process and not based on statistical tests for model fit, this was also tested with empirical measures for model fit such as Akaike information criteria (AIC), Bayesian information criteria (BIC), and likelihood ratio tests.

Besides implications for appropriate statistical estimates, CIs, and *P*-values, the amount of correlation with repeat prompting may be of interest itself, especially for study design considerations such as sample size and power calculations. The intraclass correlation coefficient (ICC) was calculated from the mixed-effects model to describe the similarity of observations within groups compared to between groups.^10^ The ICC was then used to estimate the effective sample size per grouping after accounting for correlation.^14^

In order to inform future LLM study designs, power calculations were performed using methods for cluster randomized trials with varying ICC and number of repeat prompts per group.^10^ Power calculations assumed 300 groupings of repeat prompts per arm, as in this study. Additionally, power calculations require a minimal difference to be detected, which was assumed to be 5 percentage points in the difference in mean acceptance between groups, with the lower tier group assumed to have an acceptance rate of 35%. The statistical code provided in the Supplementary Material can be adapted by researchers to improve the rigor of planned future studies.

All analyses used 2-sided hypothesis tests with a significance level of *P* < .05. Analyses were performed in R, version 4.4.0 (R Project for Statistical Computing), with code used for all analyses provided in the Supplementary Material.

## Results

As previously reported, average acceptance rate for top-tier affiliations was 38.4%, mid-tier 37.5%, and low-tier 36.7%. Simple logistic regression estimated an odds ratio of acceptance for abstracts with top-tier compared to mid-tier affiliation of 1.04 (95% CI, 1.02-1.06) which was highly statistically significant (*P* < .001). Comparing low-tier to mid-tier affiliations also demonstrated a small but highly statistically significant result (OR 0.97, 95% CI, 0.95-0.99; *P* = .002).

Table 1 shows the results from the mixed-effects model as well as the simple logistic regression model that assumes independence of observations. The comparisons between tiers go from highly statistically significant with simple logistic regression to no longer statistically significant when appropriately accounting for correlation of repeat prompts. This model with random effects for abstract and affiliation was compared to a model with a random effect for abstract alone and found to better fit the data based on AIC (181 072 vs 181 579), BIC (181 123 vs 181 621), and log likelihood (−90 531 vs −90 786; *P* < .001).

**Table 1.** Comparison of simple and random effects logistic regression.

	Simple logistic regression		Random effects logistic regression	
	Odds ratio (95% CI)	*P*	Odds ratio (95% CI)	*P*
Top-tier	1.04 (1.02-1.06)	<.001	1.07 (0.95-1.22)	.27
Low-tier	0.97 (0.95-0.99)	.002	0.94 (0.83-1.07)	.36

Mid-tier affiliations used as the reference group for all comparisons. The mixed-effects model included random effects for abstract and affiliation.

**Figure 1.**
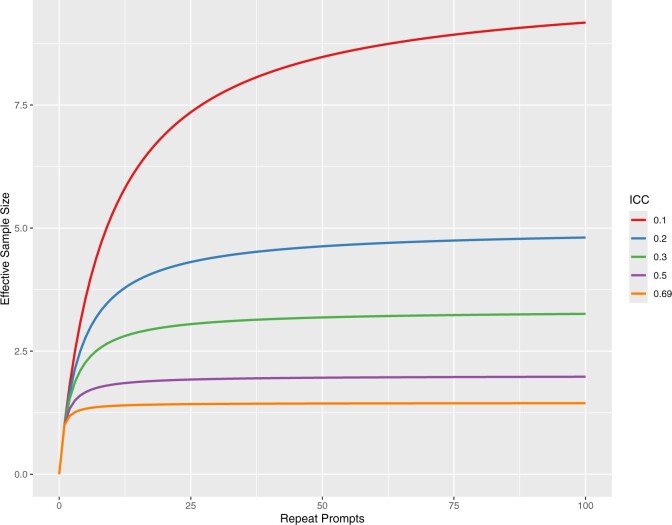
Effective sample size by intraclass correlation coefficient. Abbreviation: ICC, intraclass correlation coefficient. Repeat prompts refer to the number of repeats per grouping (ie, distinct prompt). Effective sample size is per grouping. An ICC of 0 would indicate no correlation and the effective sample size per grouping would be the same as the number within that grouping. An ICC of 0.69 was observed in this study.

**Figure 2.**
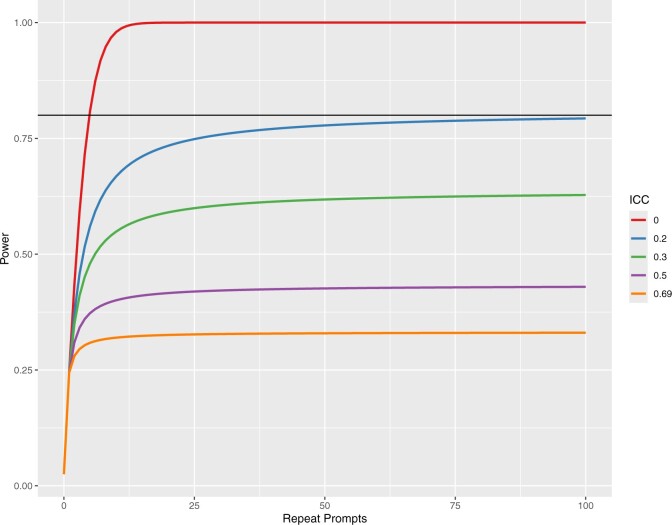
Power by intraclass correlation coefficient. Abbreviation: ICC, intraclass correlation coefficient. Repeat prompts refer to the number of repeats per grouping (ie, distinct prompt). Power calculations assume 300 groupings per arm, 5 percentage point difference between arms, and 35% acceptance rate in the comparator arm.

The ICC from the random effects model was found to be 0.69, where 0 represents no correlation and 1 represents complete correlation within groups. Using this ICC would estimate an effective sample size of 1.45 out of 250 observations per abstract-affiliation combination. Figure 1 shows the effective sample sizes with varying ICCs from 0.1 to 0.69. Power decreased from 100% to 33% when accounting for correlation in repeat prompts. Figure 2 shows the power by ICC and number of repeat prompts per group. The Supplementary Material reports results from the example using variation in prompting, with an ICC of 0.73.

## Discussion

We show that responses from repeat prompting of LLMs can be highly correlated, contrary to suggestions that repeat prompting may be thought of as independent samples.^12^ Ignoring this correlation leads to artificially narrow CIs and small *P*-values. Properly accounting for repeat prompting effectively decreased sample size by over 100-fold and decreased power by a factor of 3 in this case. The study results go from highly statistically significant to not significant with the appropriate analysis that accounts for repeat prompting, essentially nullifying the study’s conclusions.

Beyond this individual example, repeat prompting appears common in medical LLM studies.^3–7^ Repeat prompting can be helpful for measuring stability and uncertainty in model responses, and should be encouraged in study design^1^^,^^2^; however, this should not come at the expense of appropriate analysis methods. Fortunately, flexible methods exist to measure and account for this correlation in repeat prompting, such as the mixed-effects model used in the current analysis.^15^ Future studies can plan for correlation in repeat prompting using methods that have been well characterized for cluster randomized trials and other fields that deal with the same issues of data dependency.^10^^,^^14^^,^^16–18^ For instance, in this study the authors could have performed a power calculation incorporating clustering, which would have led them to include additional abstracts to achieve adequate power to answer their research question.

Large language models introduce novel evaluation and reporting complexities considering their generative and probabilistic nature, which differs from other clinical decision support tools. Given the relative recency of this field, there has been limited time to establish best practices. Current efforts include model evaluation frameworks, such as the United Kingdom AI Safety Institute’s “Inspect” open-source framework.^19^ Additionally, multiple groups are working on reporting guidelines for studies evaluating LLMs specifically in healthcare.^20^ Future efforts should include guidance on handling the probabilistic nature of LLMs, such as evaluating stability of model responses. We show that repeat prompting and prompting variations lead to substantial data dependency and should not be assumed to be independent observations, which could be incorporated into guidelines and evaluation frameworks.

This study observed an ICC of 0.69, which is relatively high, and other studies may find lower correlation within repeat prompt groupings in which models have more uncertainty. On the other hand, lower temperature settings may lead to even higher correlations since randomness would be expected to decrease as temperature decreases. Still, some models may not be completely deterministic even at temperature of zero, such as the GPT family of models. Future research could better characterize typical ICC values in LLM outputs, potentially even by type of task such as medical diagnosis, medical record summarization, patient question-answering, peer review, etc. However, the figures show that effective sample size and study power drop significantly even at lower ICC values, so accounting for correlation is nonetheless necessary for study design and statistical analyses.

## Conclusion

Rigorous evaluations of LLMs are urgently needed prior to employing this promising technology in medical settings, with stability and uncertainty in model responses an important component of any evaluation. The rapidly evolving field may outpace the ability of the scientific community to reach consensus on best practices, although there are opportunities to learn from other fields that have explored similar issues. We show that accounting for correlation in repeated prompting of LLMs is critical for valid study design and even reversed study conclusions in this case.

## Supplementary Material

ocae294_Supplementary_Data

## Data Availability

The data underlying this article are available in the online Supplementary Material.
