# Supplementary material for: Establishing best practices in large language model research: an application to repeat prompting
Source: J Am Med Inform Assoc. 2024 Dec 4;32(2):386–90. doi: 10.1093/jamia/ocae294 (PMC11756642; doi:10.1093/jamia/ocae294)
Supplement: ocae294_Supplementary_Data [file ocae294_supplementary_data.pdf]

Repeat_Prompt_Manuscript

2024-10-05

Analysis Accounting For Data Dependency

Packages

```
library(tidyverse)
```

```
## ── Attaching core tidyverse packages ──────────────────────── tidyverse 2.0.0 ──
## ✔ dplyr     1.1.4     ✔ readr     2.1.5
## ✔ forcats   1.0.0     ✔ stringr   1.5.1
## ✔ ggplot2   3.5.1     ✔ tibble    3.2.1
## ✔ lubridate 1.9.3     ✔ tidyr     1.3.1
## ✔ purrr     1.0.2
## ── Conflicts ────────────────────────────────────────── tidyverse_conflicts() ──
## ✖ dplyr::filter() masks stats::filter()
## ✖ dplyr::lag()    masks stats::lag()
## ℹ Use the conflicted package (<http://conflicted.r-lib.org/>) to force all conflicts to become errors
```

```
library(Hmisc)
```

```
##
## Attaching package: 'Hmisc'
##
## The following objects are masked from 'package:dplyr':
##
##     src, summarize
##
## The following objects are masked from 'package:base':
##
##     format.pval, units
```

```
library(lme4)
```

```
## Loading required package: Matrix
##
## Attaching package: 'Matrix'
##
## The following objects are masked from 'package:tidyr':
##
##     expand, pack, unpack
```

```
library(broom)
library(broom.mixed)
```

Affiliation Bias Data

Files from von Wedel et al. downloaded from https://osf.io/zunmx/?view_only=5d4613b6cd374e2c8ab87dcb9cd68059

I corrected the spelling of Universidad Nacional Mexico and re-leveled the tiers so mid-tier was the reference. High-tier = 1; mid-tier = 2; low-tier = 3.

The authors note that some abstract-affiliation combinations have more than 250 replicates due to overshooting during parallel execution, so I drop observations after 250 replicates per groupings as they did.

```
unis <- read_csv2("Data/Individual affiliation effects.csv") %>%
  mutate(Affiliation = replace(Affiliation, Affiliation == 'Universidad Nacional AutÃÂ³noma de MÃÂ©xico', 'Univ
ersidad Nacional Mexico'))
```

```
## ℹ Using "','" as decimal and "'.'" as grouping mark. Use `read_delim()` for more control.
```

```
## Rows: 30 Columns: 4
## ── Column specification ────────────────────────────────────────────────────────
## Delimiter: ";"
## chr (2): Affiliation, Mean acceptance
## dbl (2): hi_1mi_2low_3, Sig
##
## ℹ Use `spec()` to retrieve the full column specification for this data.
## ℹ Specify the column types or set `show_col_types = FALSE` to quiet this message.
```

```
data <- read_csv("Data/S2_1_and_2_Data_Raw_GPT_Bias.csv") %>%
  mutate(affil_tested = replace(affil_tested, affil_tested == 'Universidad Nacional AutÃ³noma de MÃ©xico', 'Unive
rsidad Nacional Mexico'))
```

```
## Rows: 232890 Columns: 11
## ── Column specification ────────────────────────────────────────────────────────
## Delimiter: ","
## chr  (1): affil_tested
## dbl (10): abstract_id, completion_id, gpt_accept, cat_intro, cat_method, cat...
##
## ℹ Use `spec()` to retrieve the full column specification for this data.
## ℹ Specify the column types or set `show_col_types = FALSE` to quiet this message.
```

```
uni_data <- left_join(data, unis, by = c("affil_tested" = "Affiliation"))

abstractAfil <- distinct(uni_data, abstract_id, affil_tested) %>%
  mutate(abstractAfil_id = as.factor(row_number()), abstract_id = as.factor(abstract_id))

afil_bias <- drop_na(uni_data, hi_1mi_2low_3) %>%
  mutate(abstract_id = as.factor(abstract_id), hi_1mi_2low_3 = as.factor(hi_1mi_2low_3)) %>%
  mutate(mi_hi_low = relevel(hi_1mi_2low_3, ref = 2)) %>%
  left_join(abstractAfil) %>%
  mutate(affil_tested = as.factor(affil_tested)) %>%
  group_by(abstractAfil_id) %>%
  slice_head(n= 250) %>%
  ungroup()
```

```
## Joining with `by = join_by(abstract_id, affil_tested)`
```

```
#description of scores

means <- group_by(afil_bias, hi_1mi_2low_3) %>%
  summarise(n = n(), accept = mean(gpt_accept))

knitr::kable(means)
```

hi_1mi_2low_3	n	accept
1	75000	0.3840667
2	75000	0.3746933
3	75000	0.3669200

```
counts <- count(afil_bias, hi_1mi_2low_3, gpt_accept) %>%
  filter(gpt_accept == 1) %>%
  mutate(n_total = 75000)

counts_hi <- filter(counts, hi_1mi_2low_3 == 1 | hi_1mi_2low_3 == 2)

counts_low <- filter(counts, hi_1mi_2low_3 == 3 | hi_1mi_2low_3 == 2)
```

Analyses

Single level

The original authors did a 2-sample proportions test. In order to compare to a multilevel model more easily, I use logistic regression instead of the proportions test.

```
#high vs mid-tier
prop.test(x = counts_hi$n, n = counts_hi$n_total, alternative = "two.sided", conf.level = 0.95)
```

```
##
##  2-sample test for equality of proportions with continuity correction
##
## data:  counts_hi$n out of counts_hi$n_total
## X-squared = 13.953, df = 1, p-value = 0.0001874
## alternative hypothesis: two.sided
## 95 percent confidence interval:
##  0.004449086 0.014297580
## sample estimates:
##    prop 1    prop 2
## 0.3840667 0.3746933
```

```
#low vs mid-tier
prop.test(x = counts_low$n, n = counts_low$n_total, alternative = "two.sided", conf.level = 0.95)
```

```
##
##  2-sample test for equality of proportions with continuity correction
##
## data:  counts_low$n out of counts_low$n_total
## X-squared = 9.6788, df = 1, p-value = 0.001864
## alternative hypothesis: two.sided
## 95 percent confidence interval:
##  0.002871403 0.012675263
## sample estimates:
##    prop 1    prop 2
## 0.3746933 0.3669200
```

```
#logistic regression, single level not accounting for clustering
sl_model <- glm(gpt_accept ~ mi_hi_low, data = afil_bias, family = binomial)
summary(sl_model)
```

```
##
## Call:
## glm(formula = gpt_accept ~ mi_hi_low, family = binomial, data = afil_bias)
##
## Coefficients:
##              Estimate Std. Error z value Pr(>|z|)
## (Intercept) -0.512134   0.007544 -67.889  < 2e-16 ***
## mi_hi_low1   0.039812   0.010643   3.741 0.000184 ***
## mi_hi_low3  -0.033319   0.010691  -3.116 0.001831 **
## ---
## Signif. codes:  0 '***' 0.001 '**' 0.01 '*' 0.05 '.' 0.1 ' ' 1
##
## (Dispersion parameter for binomial family taken to be 1)
##
##     Null deviance: 297756  on 224999  degrees of freedom
## Residual deviance: 297708  on 224997  degrees of freedom
## AIC: 297714
##
## Number of Fisher Scoring iterations: 4
```

```
sl_tidy <- tidy(sl_model)

sl_tidy$or <- exp(sl_tidy$estimate)
sl_tidy$lower <- exp(sl_tidy$estimate - 1.96*sl_tidy$std.error)
sl_tidy$upper <- exp(sl_tidy$estimate + 1.96*sl_tidy$std.error)

knitr::kable(sl_tidy)
```

term	estimate	std.error	statistic	p.value	or	lower	upper
(Intercept)	-0.5121343	0.0075437	-67.888936	0.0000000	0.5992153	0.5904207	0.6081409
mi_hi_low1	0.0398117	0.0106429	3.740685	0.0001835	1.0406148	1.0191324	1.0625501
mi_hi_low3	-0.0333187	0.0106915	-3.116385	0.0018308	0.9672303	0.9471726	0.9877127

Multilevel

The model the authors used for their reply just had a random effect for abstract ID (but of course this wouldn't capture the correlation in repeat prompting since that is by abstract/afilliation combination).

```
ml_model1 <- glmer(gpt_accept ~ mi_hi_low + (1 | abstract_id), data = afil_bias, family = binomial, nAGQ = 10)
summary(ml_model1)
```

```
## Generalized linear mixed model fit by maximum likelihood (Adaptive
##   Gauss-Hermite Quadrature, nAGQ = 10) [glmerMod]
##  Family: binomial  ( logit )
## Formula: gpt_accept ~ mi_hi_low + (1 | abstract_id)
##    Data: afil_bias
##
##      AIC      BIC   logLik deviance df.resid
## 181579.3 181620.6 -90785.7 181571.3   224996
##
## Scaled residuals:
##     Min      1Q  Median      3Q     Max
## -11.170  -0.566  -0.123   0.344  31.959
##
## Random effects:
##  Groups      Name        Variance Std.Dev.
##  abstract_id (Intercept) 7.134    2.671
## Number of obs: 225000, groups:  abstract_id, 30
##
## Fixed effects:
##             Estimate Std. Error z value Pr(>|z|)
## (Intercept) -0.99902    0.48370  -2.065   0.0389 *
## mi_hi_low1   0.07061    0.01417   4.981 6.31e-07 ***
## mi_hi_low3  -0.05909    0.01424  -4.150 3.33e-05 ***
## ---
## Signif. codes:  0 '***' 0.001 '**' 0.01 '*' 0.05 '.' 0.1 ' ' 1
##
## Correlation of Fixed Effects:
##            (Intr) m_h_l1
## mi_hi_low1 -0.014
## mi_hi_low3 -0.014  0.500
```

Just including a random effect for abstract doesn't capture the cross-randomization with affiliation, which is the unit of clustering. You have to also include affiliation as a random effect.

```
ml_model2 <- glmer(gpt_accept ~ mi_hi_low + (1 | affil_tested) + (1 | abstract_id), data = afil_bias, family = bi
nomial, nAGQ = 1)
summary(ml_model2)
```

```
## Generalized linear mixed model fit by maximum likelihood (Laplace
##   Approximation) [glmerMod]
##  Family: binomial  ( logit )
## Formula: gpt_accept ~ mi_hi_low + (1 | affil_tested) + (1 | abstract_id)
##    Data: afil_bias
##
##      AIC      BIC   logLik deviance df.resid
## 181071.5 181123.1 -90530.8 181061.5   224995
##
## Scaled residuals:
##     Min      1Q  Median      3Q     Max
## -12.676  -0.539  -0.123   0.371  34.109
##
## Random effects:
##  Groups       Name        Variance Std.Dev.
##  affil_tested (Intercept) 0.02099  0.1449
##  abstract_id  (Intercept) 7.16585  2.6769
## Number of obs: 225000, groups:  affil_tested, 30; abstract_id, 30
##
## Fixed effects:
##             Estimate Std. Error z value Pr(>|z|)
## (Intercept) -1.00176    0.28324  -3.537 0.000405 ***
## mi_hi_low1   0.07090    0.06475   1.095 0.273549
## mi_hi_low3  -0.05853    0.06461  -0.906 0.364992
## ---
## Signif. codes:  0 '***' 0.001 '**' 0.01 '*' 0.05 '.' 0.1 ' ' 1
##
## Correlation of Fixed Effects:
##            (Intr) m_h_l1
## mi_hi_low1 -0.023
## mi_hi_low3 -0.008  0.486
```

```
ml2_tidy <- tidy(ml_model2,  effects = "fixed", conf.int = TRUE, conf.method = "Wald")
ml2_tidy$or <- exp(ml2_tidy$estimate)
ml2_tidy$lower <- exp(ml2_tidy$estimate - 1.96*ml2_tidy$std.error)
ml2_tidy$upper <- exp(ml2_tidy$estimate + 1.96*ml2_tidy$std.error)

knitr::kable(ml2_tidy)
```

effect	term	estimate	std.error	statistic	p.value	conf.low	conf.high	or	lower	upper
fixed	(Intercept)	-1.0017557	0.2832387	-3.5367889	0.0004050	-1.5568934	-0.4466179	0.3672341	0.2107877	0.6397948
fixed	mi_hi_low1	0.0708964	0.0647499	1.0949261	0.2735490	-0.0560111	0.1978039	1.0734700	0.9455264	1.2187262
fixed	mi_hi_low3	-0.0585314	0.0646118	-0.9058931	0.3649924	-0.1851682	0.0681054	0.9431486	0.8309626	1.0704806

Comparing models is not really valid given we know the data was generated with clustering by abstract-affiliation combination, but will do it since used by original authors

```
anova(ml_model2, ml_model1)
```

	npar	AIC	BIC	logLik	deviance	Chisq	Df	Pr(>Chisq)
	<dbl>	<dbl>	<dbl>	<dbl>	<dbl>	<dbl>	<dbl>	<dbl>
ml_model1	4	181579.3	181620.6	-90785.65	181571.3	NA	NA	NA
ml_model2	5	181071.5	181123.1	-90530.76	181061.5	509.7912	1	7.0412e-113

2 rows

Random slope model:

```
slope <- glmer(gpt_accept ~ mi_hi_low + (1 + mi_hi_low | affil_tested) + (1 + mi_hi_low | abstract_id), data = af
il_bias, family = binomial)
summary(slope)
```

```
## Generalized linear mixed model fit by maximum likelihood (Laplace
##   Approximation) [glmerMod]
##  Family: binomial  ( logit )
## Formula: gpt_accept ~ mi_hi_low + (1 + mi_hi_low | affil_tested) + (1 +
##     mi_hi_low | abstract_id)
##    Data: afil_bias
##
##      AIC      BIC   logLik deviance df.resid
## 180553.9 180708.8 -90262.0 180523.9   224985
##
## Scaled residuals:
##     Min      1Q  Median      3Q     Max
## -15.161  -0.525  -0.123   0.373  35.057
##
## Random effects:
##  Groups       Name        Variance Std.Dev. Corr
##  affil_tested (Intercept) 0.02450  0.1565
##               mi_hi_low1  0.07318  0.2705   -0.84
##               mi_hi_low3  0.04951  0.2225   -0.86  0.74
##  abstract_id  (Intercept) 7.06732  2.6584
##               mi_hi_low1  0.06440  0.2538    0.29
##               mi_hi_low3  0.07191  0.2682   -0.01  0.35
## Number of obs: 225000, groups:  affil_tested, 30; abstract_id, 30
##
## Fixed effects:
##             Estimate Std. Error z value Pr(>|z|)
## (Intercept) -0.99314    0.30115  -3.298 0.000974 ***
## mi_hi_low1   0.05934    0.08552   0.694 0.487764
## mi_hi_low3  -0.08039    0.07978  -1.008 0.313575
## ---
## Signif. codes:  0 '***' 0.001 '**' 0.01 '*' 0.05 '.' 0.1 ' ' 1
##
## Correlation of Fixed Effects:
##            (Intr) m_h_l1
## mi_hi_low1  0.031
## mi_hi_low3 -0.064  0.464
```

Single observation

While it would be simple to not perform repeat prompting, repeat prompting does increase effective sample size even when accounting for data dependency. I've included an analysis to show what the results would be without repeat prompting.

```
set.seed(123)
single_obs <- group_by(afil_bias, abstractAfil_id) %>%
  slice_sample(n=1)

so_model <- glm(gpt_accept ~ mi_hi_low, data = single_obs, family = binomial)
summary(so_model)
```

```
##
## Call:
## glm(formula = gpt_accept ~ mi_hi_low, family = binomial, data = single_obs)
##
## Coefficients:
##               Estimate Std. Error z value Pr(>|z|)
## (Intercept) -4.055e-01  1.179e-01  -3.440 0.000581 ***
## mi_hi_low1   9.142e-15  1.667e-01   0.000 1.000000
## mi_hi_low3  -6.996e-02  1.673e-01  -0.418 0.675832
## ---
## Signif. codes:  0 '***' 0.001 '**' 0.01 '*' 0.05 '.' 0.1 ' ' 1
##
## (Dispersion parameter for binomial family taken to be 1)
##
##     Null deviance: 1207.3  on 899  degrees of freedom
## Residual deviance: 1207.0  on 897  degrees of freedom
## AIC: 1213
##
## Number of Fisher Scoring iterations: 4
```

```
so_tidy <- tidy(so_model)

so_tidy$or <- exp(so_tidy$estimate)
so_tidy$lower <- exp(so_tidy$estimate - 1.96*so_tidy$std.error)
so_tidy$upper <- exp(so_tidy$estimate + 1.96*so_tidy$std.error)

knitr::kable(so_tidy)
```

term	estimate	std.error	statistic	p.value	or	lower	upper
(Intercept)	-0.4054651	0.1178511	-3.4404855	0.0005807	0.6666667	0.5291659	0.8398963
mi_hi_low1	0.0000000	0.1666667	0.0000000	1.0000000	1.0000000	0.7213241	1.3863393
mi_hi_low3	-0.0699586	0.1673020	-0.4181576	0.6758319	0.9324324	0.6717491	1.2942783

Collapsing to a single observation per prompt would solve issues of dependency by removing dependent observations, but as you can see sample size significantly decreased and confidence intervals wider than the mixed effects model even after accounting for repeat prompts.

ICC

```
#install.packages("performance")
library(performance)

icc(ml_model2, by_group = TRUE)
```

Group	ICC
<chr>	<dbl>
affil_tested	0.00200391
abstract_id	0.68397898

2 rows

```
icc(ml_model2)
```

ICC_adjusted	ICC_unadjusted	optional
<dbl>	<dbl>	<lgl>
0.6859829	0.6857996	FALSE

1 row

Prompt Variations

The below analysis evaluates data dependency with prompt variations. The data is described in Savage et al. NPJ Digital Medicine 2024 (https://doi.org/10.1038/s41746-024-01010-1).

Briefly, we utilized different prompting strategies to evaluate GPT-3.5's and GPT-4's ability to correctly answering medical licensing questions. There were 5 prompting strategies evaluated.

The original comparison was across different prompting strategies, with one observation per model per prompt. To measure data dependency for different prompting strategies, we measure ICC for the same question and model.

```
prompting <- read_csv("data/41746_2024_1010_MOESM2_ESM-2.csv")
```

```
## Rows: 512 Columns: 12
## ── Column specification ────────────────────────────────────────────────────────
## Delimiter: ","
## chr (11): question, IR_3, AR_3, BR_3, DR_3, CoT_3, CoT_4, DR_4, IR_4, AR_4, ...
## dbl  (1): index
##
## ℹ Use `spec()` to retrieve the full column specification for this data.
## ℹ Specify the column types or set `show_col_types = FALSE` to quiet this message.
```

```
longPrompt <- select(prompting, -question) %>%
pivot_longer(-index, names_to = c("method", "model"), names_sep = "_",
                    values_to = "correct") %>%
  mutate(correct = if_else(correct == "Y", 1, 0, 0))

describe(longPrompt)
```

```
## longPrompt
##
##  4  Variables      5120  Observations
## --------------------------------------------------------------------------------
## index
##        n  missing distinct     Info     Mean      Gmd      .05      .10
##     5120        0      473        1    633.6    531.1     50.0     96.0
##      .25      .50      .75      .90      .95
##    231.5    521.0   1012.2   1382.0   1485.0
##
## lowest :    0    3    5   10   11, highest: 1586 1590 1591 1595 1597
## --------------------------------------------------------------------------------
## method
##        n  missing distinct
##     5120        0        5
##
## Value        AR   BR  CoT   DR   IR
## Frequency  1024 1024 1024 1024 1024
## Proportion  0.2  0.2  0.2  0.2  0.2
## --------------------------------------------------------------------------------
## model
##        n  missing distinct
##     5120        0        2
##
## Value         3    4
## Frequency  2560 2560
## Proportion  0.5  0.5
## --------------------------------------------------------------------------------
## correct
##        n  missing distinct     Info      Sum     Mean      Gmd
##     5120        0        2     0.73     2982   0.5824   0.4865
##
## --------------------------------------------------------------------------------
```

```
ml2 <- glmer(correct ~ model + (1 | index), data = longPrompt, family = binomial)
summary(ml2)
```

```
## Generalized linear mixed model fit by maximum likelihood (Laplace
##   Approximation) [glmerMod]
##  Family: binomial  ( logit )
## Formula: correct ~ model + (1 | index)
##    Data: longPrompt
##
##      AIC      BIC   logLik deviance df.resid
##   4547.2   4566.8  -2270.6   4541.2     5117
##
## Scaled residuals:
##     Min      1Q  Median      3Q     Max
## -7.1821 -0.3318  0.0701  0.3742  6.6510
##
## Random effects:
##  Groups Name        Variance Std.Dev.
##  index  (Intercept) 8.843    2.974
## Number of obs: 5120, groups:  index, 473
##
## Fixed effects:
##             Estimate Std. Error z value Pr(>|z|)
## (Intercept)  -0.5885     0.1551  -3.793 0.000149 ***
## model4        2.6925     0.1033  26.055  < 2e-16 ***
## ---
## Signif. codes:  0 '***' 0.001 '**' 0.01 '*' 0.05 '.' 0.1 ' ' 1
##
## Correlation of Fixed Effects:
##        (Intr)
## model4 -0.253
```

```
icc(ml2)
```

ICC_adjusted	ICC_unadjusted	optional
<dbl>	<dbl>	<lgl>
0.7288452	0.634106	FALSE

1 row

Sample Size and Power Calculations

Readers can replace the variables defined below for their own analyses.

Effective Sample Size by ICC

Effective sample size calculated using formula from: Rutterford C, Copas A, Eldridge S. Methods for sample size determination in cluster randomized trials. Int J Epidemiol. 2015;44(3):1051–67.

```
x1 <- 0  # lowest cluster size considered
x2 <- 100  # highest cluster size considered

# Generating sequence for cluster sizes
x_values <- seq(from = x1, to = x2, by = 1)

#effective sample is cluster size/design effect
calc_es <- function(x, ro) {
  es <- x/(1 + ((x-1)*ro))
  return(es)
}
# Different values of ro to explore
ro_values <- c(0.1, 0.2, 0.3, 0.5, 0.69)

# Prepare data for plotting
plot_es_data <- data.frame()
for (ro in ro_values) {
  es_values <- sapply(x_values, calc_es, ro)
  temp_data <- data.frame(cluster_size = x_values, es = es_values, ro = ro)
  plot_es_data <- rbind(plot_es_data, temp_data)
}


# Plotting
ggplot(plot_es_data, aes(x = cluster_size, y = es, color = as.factor(ro))) +
  geom_line(linewidth = 1) +
  labs(color = 'ICC', x = 'Repeat Prompts', y = 'Effective Sample Size') +
  scale_color_brewer(palette = "Set1")
```

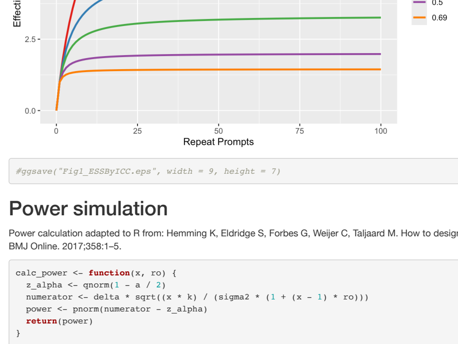


```
#ggsave("Fig1_ESSByICC.eps", width = 9, height = 7)
```

Power simulation

Power calculation adapted to R from: Hemming K, Eldridge S, Forbes G, Weijer C, Taljaard M. How to design efficient cluster randomised trials. BMJ Online. 2017;358:1–5.

```
calc_power <- function(x, ro) {
  z_alpha <- qnorm(1 - a / 2)
  numerator <- delta * sqrt((x * k) / (sigma2 * (1 + (x - 1) * ro)))
  power <- pnorm(numerator - z_alpha)
  return(power)
}

# Constants
k <- 300  # number of clusters per arm; in this case it would be 30 abstracts * 10 affiliations in each tier
p0 <- 0.35  # proportion in control arm
p1 <- 0.4  # proportion in intervention arm
a <- 0.05  # alpha level
delta <- p1 - p0
sigma0 <- p0 * (1 - p0)
sigma1 <- p1 * (1 - p1)
sigma2 <- sigma0 + sigma1
x1 <- 0  # lowest cluster size considered
x2 <- 100 # highest cluster size considered

# Generating sequence for cluster sizes
x_values <- seq(from = x1, to = x2, by = 1)

# Different values of ro to explore
ro_values <- c(0, 0.2, 0.3, 0.5, 0.69)

# Prepare data for plotting
plot_data <- data.frame()
for (ro in ro_values) {
  power_values <- sapply(x_values, calc_power, ro)
  temp_data <- data.frame(cluster_size = x_values, Power = power_values, ro = ro)
  plot_data <- rbind(plot_data, temp_data)
}

# Plotting
ggplot(plot_data, aes(x = cluster_size, y = Power, color = as.factor(ro))) +
  geom_line(linewidth = 1) +
  labs(color = 'ICC', x = 'Repeat Prompts', y = 'Power') +
  scale_color_brewer(palette = "Set1") +
  geom_hline(yintercept = 0.8)
```

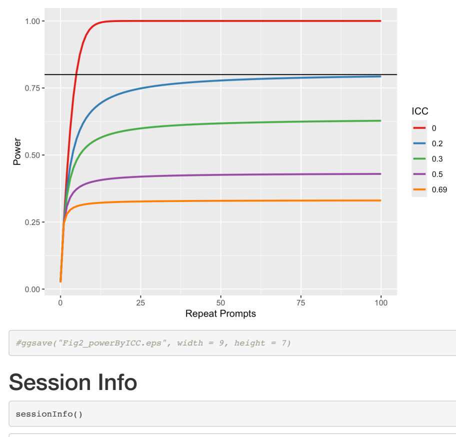


```
#ggsave("Fig2_powerByICC.eps", width = 9, height = 7)
```

Session Info

```
sessionInfo()
```

```
## R version 4.4.0 (2024-04-24)
## Platform: x86_64-apple-darwin20
## Running under: macOS Ventura 13.6.9
##
## Matrix products: default
## BLAS:   /Library/Frameworks/R.framework/Versions/4.4-x86_64/Resources/lib/libRblas.0.dylib
## LAPACK: /Library/Frameworks/R.framework/Versions/4.4-x86_64/Resources/lib/libRlapack.dylib;  LAPACK version 3.
12.0
##
## locale:
## [1] en_US.UTF-8/en_US.UTF-8/en_US.UTF-8/C/en_US.UTF-8/en_US.UTF-8
##
## time zone: America/Los_Angeles
## tzcode source: internal
##
## attached base packages:
## [1] stats     graphics  grDevices utils     datasets  methods   base
##
## other attached packages:
##  [1] performance_0.11.0  broom.mixed_0.2.9.5 broom_1.0.5
##  [4] lme4_1.1-35.3       Matrix_1.7-0        Hmisc_5.1-3
##  [7] lubridate_1.9.3     forcats_1.0.0       stringr_1.5.1
## [10] dplyr_1.1.4         purrr_1.0.2         readr_2.1.5
## [13] tidyr_1.3.1         tibble_3.2.1        ggplot2_3.5.1
## [16] tidyverse_2.0.0
##
## loaded via a namespace (and not attached):
##  [1] tidyselect_1.2.1   farver_2.1.2       fastmap_1.2.0      digest_0.6.35
##  [5] rpart_4.1.23       timechange_0.3.0   lifecycle_1.0.4    cluster_2.1.6
##  [9] magrittr_2.0.3     compiler_4.4.0     rlang_1.1.4        sass_0.4.9
## [13] tools_4.4.0        utf8_1.2.4         yaml_2.3.8         data.table_1.15.4
## [17] knitr_1.47         labeling_0.4.3     htmlwidgets_1.6.4  bit_4.0.5
## [21] RColorBrewer_1.1-3 withr_3.0.0        foreign_0.8-86     nnet_7.3-19
## [25] grid_4.4.0         fansi_1.0.6        colorspace_2.1-0   future_1.33.2
## [29] globals_0.16.3     scales_1.3.0       MASS_7.3-60.2      insight_0.19.10
## [33] cli_3.6.2          rmarkdown_2.27     crayon_1.5.2       generics_0.1.3
## [37] rstudioapi_0.16.0  tzdb_0.4.0         minqa_1.2.6        cachem_1.1.0
## [41] splines_4.4.0      parallel_4.4.0     base64enc_0.1-3    vctrs_0.6.5
## [45] boot_1.3-30        jsonlite_1.8.8     hms_1.1.3          bit64_4.0.5
## [49] Formula_1.2-5      htmlTable_2.4.2    listenv_0.9.1      jquerylib_0.1.4
## [53] glue_1.7.0         parallelly_1.37.1  nloptr_2.0.3       codetools_0.2-20
## [57] stringi_1.8.4      gtable_0.3.5       munsell_0.5.1      pillar_1.9.0
## [61] furrr_0.3.1        htmltools_0.5.8.1  R6_2.5.1           vroom_1.6.5
## [65] evaluate_0.23      lattice_0.22-6     highr_0.11         backports_1.5.0
## [69] bslib_0.7.0        Rcpp_1.0.12        gridExtra_2.3      nlme_3.1-164
## [73] checkmate_2.3.1    xfun_0.44          pkgconfig_2.0.3
```
